# Effects of use of metformin or combination of metformin and pioglitazone on oxidative stress in type 2 diabetes mellitus

**DOI:** 10.5937/jomb0-50062

**Published:** 2025-03-21

**Authors:** Temiz Gençoğlu Ayşe Tuğçenur, Kalkan Emra Asfuroğlu, Oğuzhan Zengin, Özcan Erel, İhsan Ateş

**Affiliations:** 1 Ankara Bilkent City Hospital, Department of Internal Medicine, Ankara, Turkey; 2 Ankara Bilkent City Hospital, Department of Biochemistry, Ankara, Turkey

**Keywords:** type 2 diabetes mellitus, metformin, pioglitazone, oxidative stress, dijabetes melitus tip 2, metformin, pioglitazon, oksidativni stres

## Abstract

**Background:**

We aimed to examine how oxidative stress changes after treatment and its relationship with metabolic parameters in patients diagnosed with Type 2 Diabetes Mellitus treated with metformin alone or with a combination of metformin and pioglitazone.

**Methods:**

The study population consisted of a total of 60 patients, including the group diagnosed with Type 2 Diabetes Mellitus (T2DM) who received metformin 2x1000 mg (n=30) and the other group who received metformin with pioglitazone 2x15/1000 mg (n=30). Serum IMA (ischemia-modified albumin), TAS (total antioxidant status), TOS (total oxidant status) and thiol-disulphide homeostasis were measured before and after 12 weeks of treatment.

**Results:**

No significant change was detected in Native sulfhydryl (SH) and Total SH levels in both Metformin and combination groups. IMA levels increased significantly in both drug groups (p=0.03 and p=0.01, respectively). Although the TAS level increased in both groups, no significant change was detected. While the TOS and oxidative stress index (OSI) index decreased significantly in the combination group (p<0.001), the decrease in the metformin group was not significant. No significant difference was detected between Native SH, Total SH, Disulfide, IMA, TAS, TOS levels and OSI index changes of both drug groups.

**Conclusions:**

We found that the use of anti-diabetic drugs did not have a significant effect on oxidative stress.

## Abstract (sr)

**Uvod:**

Cilj nam je bio da ispitamo kako se oksidativni stres menja nakon terapije i njegovu povezanost sa metabo - ličkim parametrima kod pacijenata sa dijagnozom dijabetes melitusa tipa 2 koji su lečeni samo metforminom ili kombinacijom metformina i pioglitazona.

**Metode:**

Studiju je činilo ukupno 60 pacijenata, uključujući grupu sa dijagnozom dijabetes melitus tip 2 (T2DM) koja je primala metformin 2x1000 mg (n=30) i drugu grupu koja je primala kombinaciju metformina i pioglitazona 2x15/1000 mg (n=30). Serumski nivoi IMA (albumin modifikovan ishemijom), TAS (ukupni antioksidativni status), TOS (ukupni oksidativni status) i homeostaza tioldisulfida mereni su pre i posle 12 nedelja terapije.

**Rezultati:**

Nije detektovana značajna promena u nivoima nativnog sulfhidrila (SH) i ukupnog SH u obe grupe: metformin i kombinovana. Nivoi IMA su značajno porasli u obe grupe lekova (p=0,03 i p=0,01, respektivno). Iako je nivo TAS porastao u obe grupe, nije primećena značajna promena. Dok su TOS i indeks oksidativnog stresa (OSI) značajno opali u grupi sa kombinacijom lekova (p<0,001), opadanje u grupi sa metforminom nije bilo značajno. Nije otkrivena značajna razlika između promena nivoa nativnog SH, ukupnog SH, disulfida, IMA, TAS, TOS i indeksa OSI izme|u obe grupe lekova.

**Zaključak:**

Utvrdili smo da upotreba antidijabetičkih lekova nije imala značajan uticaj na oksidativni stres.

## Introduction

Diabetes mellitus is a disease with high morbidity and mortality that plays a role in the etiopathogenesis of cardiovascular diseases. The role of oxidative stress in developing diabetes and its complications was first observed experimentally in 1982 [1]. In current studies, it has been reported that free radicals and lipid peroxidation increase significantly in experimental diabetic rats and diabetics and that oxidative stress plays a role in the pathogenesis and progression of diabetes [2]. Type 2 diabetes treatment aims to reduce and delay related complications and mortality. For this purpose, lifestyle changes, reduction of blood glucose and lipids to recommended target levels, blood pressure control, use of antiplatelet agents and smoking cessation should be focused. Although anti-diabetic agents help to reduce oxidative stress indirectly by lowering blood sugar levels, they also directly reduce oxidative stress by upregulating the antioxidant system and suppressing free radicals.

In situations such as ischemia, hypoxia, acidosis, and instances of heightened free oxygen radicals, Ischemia-Modified Albumin (IMA) levels increase [3] [4]. Total Oxidant Capacity (TOS) serves as an indicator of oxidative stress throughout the entire body. Although antioxidant molecules can be measured separately, it is recommended to measure Total Antioxidant Capacity (TAS) since endogenous and exogenous antioxidant molecules in the body act synergistically together. Thiols or mercaptans (C-SH) are organic sulfur derivatives containing sulfhydryl (-SH) groups in their active sites. Thiols neutralise reactive oxygen products by forming disulfide bonds (-S-S-) in the organism. In other words, in oxidative damage, thiol levels decrease while disulfide levels increase [5].

Our study determined how oxidative stress changes after treatment and its relationship with metabolic parameters in patients diagnosed with Type 2 Diabetes Mellitus treated with metformin alone or in combination with pioglitazone.

## Materials and methods

Our research was planned as a single-centre, prospective, and observational study. This research was conducted between June 15, 2020 and November 15, 2020, at the internal medicine clinic of the hospital to which the authors are affiliated. The study was planned following the decisions of the Declaration of Helsinki and patient rights regulations. The study was approved by the Clinical Research Ethics Committee of the Hospital with approval number E1/866/2020.

Patients aged 18–65, of both genders, diagnosed with Type 2 Diabetes Mellitus (T2DM) and prescribed either metformin alone or a combination of metformin and pioglitazone, as indicated, were included in the study. Patients with acute kidney injury or chronic kidney injury, GFR<60 mL/min, liver failure, hypertension, history of coronary artery disease, history of cerebrovascular accident, history of peripheral artery disease, chronic pulmonary disease (chronic obstructive pulmonary disease) and malignancy were excluded from the study. According to the New York Heart Association’s criteria, cases in classes I-IV for congestive heart failure and patients receiving immunosuppressive therapy were excluded from the study. It was planned to exclude from the study patients who had acute renal failure, hypervolemia, increased transaminases, diabetic ketoacidosis/lactic acidosis/hyperosmolar non-ketotic coma, or fractures secondary to osteoporosis during follow-up.

The study population consisted of 60 patients, including the group in which metformin 2000 mg was given (n=30) and the pioglitazone -metformin 30/2000 mg combination was given (n=30) with the diagnosis of T2DM. Patients’ age, gender, comorbidities, medications, smoking and alcohol use were recorded.

Urea, creatinine, sodium, potassium, albumin, complete urine analysis, complete blood count, spot urine protein, spot urine creatinine, spot urine microalbumin level, HbA1c level, thyroid stimulating hormone (TSH), free T3, free T4, total cholesterol, Triglyceride, low-density lipoprotein (LDL) and high-density lipoprotein (HDL) levels were measured from routine blood samples at the beginning of the treatment and the 3rd month after the 12-week follow-up period. The glomerular filtration rate was calculated using the CKD-EPI formula. Fresh urine samples were taken in the morning to measure microalbuminuria concentration. Serum IMA, TAS, TOS and thiol/disulfide levels were evaluated specifically for the study. Blood samples were taken after 12 hours of fasting. The blood samples taken were studied in the Central Laboratory of the Hospital.

All patients received counselling on lifestyle modification for T2DM, including diet and exercise. The hemogram values of the patients were studied with the Mindray BC-6800 (Medical International Limited, Shenzhen, China) device. HbA1c and lipid parameters were analysed with an ARCHITECT c16000 (Abbott Laboratories, Abbott Park, Illinois, USA) model device. HbA1c was studied by immuno-turbidimetric methods. Lipid levels were analysed using the colorimetric enzyme test method. Spot urinalysis was performed with the ARCHITECT c4000 (Abbott Laboratories, Abbott Park, Illinois, USA) device.

TAS and TOS levels were measured with a Relassay brand commercial elisa kit (Relassay, Turkey). It was run fully automatically on a Mindray BS300 (Medical International Limited, Shenzhen, China) device, and the results were expressed in millimolar trolox (mmol Trolox equivalent/L) for TAS and in micromolar hydrogen peroxide equivalent (μmol H_2_O_2_Eqv./L) for TOS. Thiol / Disulfide Balance was measured by automatic spectrophotometric method on Siemens ADVIA 1800 (Siemens Healthineers AG, Erlangen, Germany) device and expressed as μmol/L. IMA level was measured on the Siemens ADVIA 1800 (Siemens Healthineers AG, Erlangen, Ger many) device, and the results were expressed as absorbance units (ABSU) (kyn).

OSI was calculated with the formula »OSI (arbitrary unit)=TOS (μmol H_2_O_2_ equiv./L)/TAS (mmol Trolox equiv./L)« Since there is no standard value and range for the OSI level, it was used only for comparison between groups.

### Statistical analysis

Statistical evaluation was performed using the Statistical Package for Social Sciences (SPSS) for Windows 20 (IBM SPSS Inc., Chicago, IL) program. The normal distribution of the data was evaluated with the Shapiro-Wilk test. Numerical variables with normal distribution were shown as mean±standard deviation, and numerical variables with non-normal distribution were shown as median (interquartile range =IQR). Categorical variables were expressed as numbers and percentages. Chi-Square and Fisher’s Exact test were used to compare categorical data. Independent t-test was used to compare normally distributed numerical variables between two groups, and the Whitney U test was used to compare non-normally distributed numerical variables. T and Wilcoxon’s tests were used in dependent samples to compare initial evaluation and control numerical variables in the entire population. The relationship of oxidative parameters with other variables was evaluated using Spearman’s correlation analysis. The effects of drug groups on changes in oxidative parameters were examined using repeated measures of ANOVA. In statistical analysis, a p-value of <0.05 was considered significant.

## Results

When the clinical and demographic characteristics of the patient groups were examined, male gender was predominant in the combination group (70%) and female gender (63.3%) in the metformin group (p = 0.01). No significant difference was found between the groups in terms of smoking and alcohol use (p = 0.7, p = 0.6, respectively). Again, when the presence of additional comorbidities and additional medication use were compared between the groups, no significant difference was detected (p = 0.45, p = 0.2, respectively). Demographic characteristics are shown in detail in Table 1.

**Table 1:** Sociodemographic characteristics of the * p<0.05 significant value<br>Abbreviations: ASA, acetyl salicylic acid.

		Metformin<br>N=30 +<br>mean±SS		Metformin+Pioglitazon<br>N=30<br>mean±SS		P-value
		N	%	N	%	
Age		51.23±8.65		50.77±7.35		p= 0.82
Gender, n (%)	Woman	19	63.3	9	30	p= 0.01*
	Man	11	36.6	21	70	
Smoke, n (%)	No	24	80	25	83.3	p= 0.7
	Yes	6	20	5	16.6	
Alcohol, n (%)	No	29	96.6	27	90	p= 0.6
	Yes	1	3.3	3	10	
Comorbidity, N (%)	No	23	76.6	27	90	p= 0.45
	Hypothyroidism	5	16.6	1	3.3	
	Astma	1	3.3	1	3.3	
	Hyperlipidemia	1	3.3	1	3.3	
Additional drug use, n (%)	No	28	93.3	29	96.6	p=0.2
	Statin group	1	3.3	0	0	
	ASA	1	3.3	0	0	

In patients receiving metformin treatment (n=30), a significant decrease was detected in FBG, urea, HbA1c, and UPR/UKR values 12 weeks after treatment, while a significant increase was detected in HDL levels (p<0.05). In patients receiving Metformin and Pioglitazone combination therapy (n=30), a significant decrease was detected in FBG, HbA1c, and Triglyceride values 12 weeks after treatment (p<0.05). It is shown in detail in Table 2.

**Table 2:** Pre-treatment and post-treatment biochemistry and spot urine parameters of the treatment groups.

	metformin<br>first application<br>n=30	metformin<br>second application<br>n=30	p	metformin+pioglitazon-<br>first application<br>n=30	metformin+pioglitazon-<br>second application<br>n=30	p
FBG (mmol/L)	120.56.7 (84.7)	113 6.3 (1.7)	p<0.001*	11.9 (7.2)	7.2 (3.0)	p<0.001*
Urea (mmol/L)	1.7 (0.2)	1.6 (0.4)	p=0.01*	1.6 (0.6)	1.7 (0.6)	p=0.44
Creatinine (mmol/L)	68.08±12.3	66.3±13.2	p=0.21	76.04±15.03	72.5±16.7	p=0.21
HbA1c (%)	7.05 (2.6)	6.9 (1)	p<0.001*	9.85 (3.2)	7.4 (2)	p<0.001*
Triglyceride 552 (mmol/L)	10.7 (103)	9.8 (7.7)	p=0.34	12.1 (12.3)	8.0 (9.9)	p=0.02*
LDL (mmol/L)	7.1 (2.5)	6.5±1.9	p=0.57	6.6 (2.8)	6.4±1.7	p=0.90
HDL (mmol/L)	2.5 (1.5)	2.6 (0.9)	p=0.06*	2.3 (0.7)	2.4 (1.1)	p=0.30
Non-HDL (mmol/L)	9.1±2.1	8.5±2.1	p=0.1	9.1±2.4	8.4±2	p=0.11
UPR/UCR (mg/g cr)	76.77 (50)	65.5 (41)	p=0.02*	74.35 (86.33)	70 (42.5)	p=0.06
UMA/UCR (mg/g cr)	6.65 (14. 4)	4.75 (9. 45)	p=0.83	9.00 (23.75)	7.95 (16.15)	p=0.09

In the group receiving metformin treatment, no relationship was found between oxidative stress parameters and age, gender, HbA1c level and FBG level change. It is shown in detail in Table 3.

**Table 3:** Effect of age, gender, HbA1c and fasting blood glucose level change on oxidative stress change in the metformin group. Spearman correlation analysis<br>*p<0.05 statistical significance

		HbA1c Level<br>Difference	FBG Level<br>Difference	c	Gender
Native Thiol Level<br>Difference	r	-0.205	-0.032	-0.258	-0.188
	p	0.278	0.866	0.168	0.320
Total Thiol Level<br>Difference	r	-0.237	-0.018	-0.276	-0.204
	p	0.208	0.926	0.140	0.280
Disulfide Level<br>Difference	r	-0.282	0.021	-0.297	-0.116
	p	0.131	0.912	0.111	0.542
IMA Level<br>Difference	r	-0.082	-0.156	0.075	-0.140
	p	0.665	0.411	0.695	0.461
TAS Level<br>Difference	r	-0.184	-0.319	-0.197	-0.144
	p	0.330	0.086	0.297	0.448
TOS Level<br>Difference	r	0.056	0.131	-0.286	0.164
	p	0.769	0.490	0.125	0.387
OSI Level<br>Difference	r	0.148	0.241	-0.228	0.148
	p	0.436	0.200	0.226	0.436

In the group receiving metformin and pioglitazone combination therapy, no relationship was found between the change in oxidative stress markers, HbA1c, and FBG levels. A negative correlation was detected between the number of female patients and native thiol change (r=-0.576, p=0.001), total thiol change (r=-0.567, p=0.001), disulphide change (r=-0.517, p=0.003), IMA change (r=-0.551, p=0.002). TAS change (r =-0.484, p=0.007). No relationship was found between TOS and OSI index changes and gender. A negative correlation was detected between age and TOS level change (r=-0.590. p=0.001) and OSI index level change (r=-0.582. p=0.001). No relationship was found between changes in other oxidative stress markers and age. It is shown in detail in Table 4.

**Table 4:** Effect of age, gender, HbA1c and fasting blood sugar level change on oxidative stress change in the metformin and pioglitazone combination group. Spearman correlation analysis<br>*p<0.05 statistical significance

		HbA1c Level Difference	FBG Level Difference	Age	Gender
Native Thiol Level Difference	r	0.085	-0.051	-0.109	-0.576**
	p	0.655	0.787	0.566	** 0.001 **
Total Thiol Level Difference	r	0.091	-0.061	-0.071	-0.567**
	p	0.632	0.747	0.711	** 0.001 **
Disulfide Level Difference	r	0.106	-0.006	-0.088	-0.517**
	p	0.578	0.976	0.644	** 0.003 **
Ima Level Difference	r	-0.129	-0.265	0.009	-0.551**
	p	0.496	0.157	0.962	** 0.002 **
Tas Level Difference	r	0.098	-0.95	-0.162	-0.484**
	p	0.605	0.302	0.392	** 0.007 **
Tos Level Difference	r	0.189	0.262	-0.590**	-0.008
	p	0.317	0.162	** 0.001 **	0.965
Osi Level Difference	r	0.168	0.290	-0.582**	0.197
	p	0.375	0.120	** 0.001 **	0.296

No significant change was detected in Native SH and Total SH levels in both Metformin and combination groups (p=0.22, p=0.12 and p=0.72, p=0.57, respectively). IMA levels increased significantly in both drug groups (p=0.03 and p=0.01, respectively). Although the TAS level increased in the metformin and combination group, no significant change was detected (p=0.11, p=0.16). While TOS decreased significantly in the combination group (p<0.001), the decrease in the metformin group was not significant (p=0.18). While the OSI index reduced considerably in the combination group (p<0.001), the reduction was not substantial in the metformin group (p=0.11). No significant difference was detected between Native SH, Total SH, Disulfide (SS), IMA, TAS, TOS levels and OSI index changes of both drug groups (p=0.66, p=0.63, p=0.56, p=0.43, p=0.84, p=0.11, p=0.12, respectively). It is shown in detail in Table 5.

**Table 5:** Relationship between oxidative stress parameters and drugs in both groups before and after treatment. Δp: Comparison of pre- and post-treatment changes between treatment groups<br>*p<0.05 statistical significance

	Metformin		p	Metformin+Pioglitazon		p	Δp
	first application	Control		first	Control		
	N=30<br>Mean±SS/<br>interquartile<br>range =IQR	N=30<br>Mean±SS/<br>interquartile<br>range =IQR		application<br>N=30Mean±SS/<br>interquartile<br>range =IQR	N=30<br>Mean±SS/<br>interquartile<br>range =IQR		
NATIVE SH [Native Thiol (mmol/L)]	318.37±46.99	327.94±34.74	p=0.22	305.66±50.91	309.45±39.44	p=0.72	p=0.66
TOTAL SH [Total Thiol (mmol/L)]	358.69±62.02	374.83±44.98	p=0.12	342.15±66.97	349.94±52.03	p=0.57	p=0.63
SS [Disulfide (mmol/L)]	20.16±7.70	23.95 (7.29)	p=0.21*	18.24±8.96	19.97 (10.29)	p=0.33	p=0.56
IMA [Ischemia Modified Albumin (ng/mL)]	0.70±0.05	0.74 (0.06)	p=0.03*	0.68±0.07	0.73 (0.1)	p=0.01*	p=0.43
TAS [Total Antioxidant Status (mmol Trolox equivalent/L]	1.52±0.2	1.57±0.22	p=0.11	1.39±0.2	1.45±0.22	p=0.16	p=0.84
TOS [Total Oksidant Status (mmol Eq/L)]	11.05 (3.5)	8.15 (7.12)	p=0.18	17.19 (5.1)	8.15 (3.15)	p<0.001*	p=0.11
OSI (arbitrary unit)	7.48 (2.95)	5.4 (4.24)	p=0.11	7.56 (3.68)	5.44 (2.18)	p<0,.001*	p=0.12

## Discussion

We designed our study to examine how oxidative stress changes after treatment and its relationship with metabolic parameters in patients diagnosed with Type 2 Diabetes Mellitus.

In our study, SH and TT were significantly lower in the combination group with poor glycemic control in pre-treatment oxidative stress parameters compared to the metformin group, while the SS level was also lower. In the study of Özler et al. [6], it was shown that the amount of SS in the cord blood of babies born to obese and gestational diabetic mothers increased, and the Native SH/Total SH ratios decreased. In the same study, increased SS in cord blood and a decreased Native SH/Total SH ratio were associated with poor perinatal outcomes. In another study comparing 125 prediabetic patients and 125 healthy volunteers, Total SH and Native SH values of prediabetic patients were significantly lower compared to the control group, while SS values were significantly higher [7].

The reason why SS values, which are expected to increase due to decreased Native SH and Total SH values, decreased in our study is that the chemically established disulfide bonds in individuals in case of oxidative stress are broken by another antioxidant defence mechanism that develops in the body as the oxidative stress level increases. It may also be explained by the nature of TH/SS homeostasis, which is that disulfide bonds are in a constant state of construction and destruction in the case of oxidative stress [8].

In our study, the IMA level was unexpectedly lowest in the combination group with the worst glycemic control. The first study on IMA was conducted by Piwowar et al. [9], who found that the IMA level in patients with T2DM was higher than in healthy individuals. In the study conducted by Chawla et al. [10] to determine the oxidative stress level in diabetic patients, IMA levels were higher in patients with poor glycemic control than in the group with good glycemic control and were correlated with HbA1c levels. In a cross-sectional study by Ghosh et al. [11], in which the IMA level was evaluated in the prediction of complications in T2DM patients when 100 diabetic patients with 78 complications were compared with 100 healthy control groups, IMA levels were found to be significantly higher in the complicated T2DM group. Ghareghani O et al. [12] emphasised in their review ‘IMA level indicates the advanced stage of DM complications.’

In our study, the lower IMA level in the combination group than in the metformin group may be because diabetic complication groups were not separated. Additionally, when looking at the literature, there are very contradictory results regarding IMA. In the cross-sectional study of Ate et al. [13], IMA levels were lower in the insulin-resistant group than in the non-insulin-resistant group and overweight groups. In a study conducted by Dahiya et al. [14], no significant difference was found between the IMA level of individuals with T2DM without vascular complications and healthy individuals. When we evaluate the existing studies in the literature and consider our study, it is evident that the IMA level is affected by different mechanisms, like peptic ulcer, other than glycemic control, that need to be elucidated.

In our study, although there was a decrease in serum TOS levels in both drug groups, we found a significant decrease in the metformin-pioglitazone combination group. Still, we did not detect an important difference between the groups when compared. We found that the OSI rate decreased significantly in the metformin-pioglitazone combination group. However, it decreased in both groups, but there was no significant difference between them. We thought there would have been an essential result if there were more patients.

Although there are similar studies in the literature, we have not come across any study showing the change in TOS and TAS levels in both drug groups after treatment, which provides more valuable information than examining and evaluating oxidant and antioxidant enzymes one by one, as they reflect the general oxidative status in the body. To the best of our knowledge, our study is a first in this regard. With rapid developments in diabetes treatment in the medical world, costs are increasing. The effectiveness and safety of both drugs evaluated in our study have been proven in many studies over the years, and their lower price compared to many other anti-diabetic drugs in terms of cost-effectiveness is a reason for preference.

This study had some limitations. First, although this study’s prospective and comparative design were advantages, it included data from a single centre. Another limitation is that it consists of relatively few patients; their compliance with diet and lifestyle change recommendations is unknown, and their weight is not monitored. Patients were not differentiated according to the presence of diabetic complications. In addition, many parameters indicate oxidant and antioxidant balance, and we were able to evaluate only some of them in our study. The fact that these parameters are related to different factors may have caused us to obtain other results.

## Conclusion

As a result, it was determined that metformin or metformin-pioglitazone combinations did not significantly affect oxidative stress in diabetic patients. To more clearly reveal the effects of metformin or metformin-pioglitazone combinations on oxidative stress, future randomised controlled studies with larger sample sizes, longer follow-up periods, and more parameters are needed.

## Dodatak

### Conflict of interest statement

All the authors declare that they have no conflict of interest in this work.
